# Evaluation of staff satisfaction after the implementation of a daily goals sheet in the routine work of an oral outpatient department and its influence on work efficiency

**DOI:** 10.1186/s12913-022-08028-9

**Published:** 2022-05-17

**Authors:** Wei Zhang, Lizhi Ren, Xiaojing Wang, Qianqian Wang, Xiaohong Zhang, Weili Li, Bin Zhao, Xiuyun Ren, Bing Li, Haiyun Qin, Xuejun Ge, Fang Zhang, Xiangyu Wang, Xiaorui Meng, Feiyan Yu

**Affiliations:** 1 Department of Oral Medicine, Shanxi Medical University School and Hospital of Stomatology, No. 63, New South Road, Yingze District, Shanxi 030001 Taiyuan, People’s Republic of China; 2 Shanxi Medical University School and Hospital of Stomatology, Clinical Medical Research Center of Oral Diseases of Shanxi Province, Taiyuan, 030001 Shanxi China; 3 Department of Oral Medicine, Shanxi Medical University School and Hospital of Stomatology, No. 63, New South Road, Yingze District, Taiyuan, 030001 Shanxi People’s Republic of China

**Keywords:** Dental clinic, Daily goals sheet, Work efficiency, Satisfaction, Plan-Do-Check-Act

## Abstract

**Background:**

In March 2021, the supervision group of our hospital inspected the daily work of the outpatient department in the branch and found many problems in the process, such as an excessive number of daily check-up forms, nurses’ confusion regarding the daily check-up process, and the omission of daily check-up items. Therefore, focusing on these problem, our hospital established a quality improvement team to conduct a status survey and perform this study. This study evaluated the feasibility, availability and sustainability of using a daily goals sheet in the routine work of a stomatological outpatient department and investigated the satisfaction of the nursing staff with the sheet.

**Methods:**

After determining the theme of this study through the status survey, 60 nurses were randomly selected and divided into an experimental group and a control group by a random grouping method. Then, the study was divided into two stages: Applying the PDCA cycle method and following the MECE (Mad Exclusive, Collectively Exhaustive) principle to design, manufacture and apply the daily goals sheet. After the expert group performed Stage one, an analysis of work efficiency and routine omissions and a staff satisfaction survey were carried out. The results of the groups either using the daily goals sheet (*n* = 30) or not (*n* = 30) were analysed and compared.

**Results:**

The average work time of the daily goals sheet group was 15.20 ± 1.70 min, and that of the nondaily goals sheet group was 25.30 ± 2.70 min (*P* < 0.001). The omission rate was 0% in the daily goals sheet group and 16.67% in the nondaily goals sheet group. Staff satisfaction with the use of the daily goals sheet was high.

**Conclusion:**

The daily goals sheet can make routine work more efficient and convenient in a stomatological outpatient department. It is recommended for use in stomatological outpatient departments or hospitals.

## Introduction

Since its publication in 2009, the World Health Organization’s Surgical Safety Checklist has been impressively successful and widely used in medicine [1]. Surgical safety checklists are used to facilitate teamwork, share important clinical information, and effectively prevent errors or omissions [2]. The implementation of surgical safety checklists worldwide has resulted in significant reductions in operation-related complications and mortality [3].

In oral outpatient medical activities, there are few reports on oral professional medical complications. This may be related to insufficient data collection or to the reluctance of practitioners to report incidents for fear of losing clients or revenue. Injury (10%), medical emergency (6%), aspiration or ingestion (4%), adverse reactions (4%), and dental placement or extraction errors (2%) are the major complications reported in the literature [4]. Therefore, in 2018, a safety checklist for oral and maxillofacial surgery was designed and applied to clinical work. Statistical analysis of medical staff satisfaction and postoperative adverse events showed that staff had a high degree of satisfaction with the use of the checklist. In addition, team communication was effectively improved, the pressure level during the operation was reduced, and the frequency of postoperative adverse events was significantly reduced[5]. Check-ups are commonly used in a range of complex medical activities, including procedures for validating the safety of surgery, implementing hospital sensation control procedures, and routine care in the hospital [1, 6, 7]. In healthcare, checklists have unique advantages, including mobility, flexibility and visibility of work [8]. However, checklists do not receive enough attention in general hospitals or oral clinics, which may be due to the variable workflows or the inability to identify appropriate checklists [9, 10]. The daily goals sheet is designed in accordance with the principle of the checklist, which pays more attention to the check and recording of multiple tasks in a day. Since there are few reports on the application of checklists or daily goals sheet in the routine work of stomatology outpatient departments, this paper discusses their applications and advantages.

The routine work of the dental clinic is equally important to routine medical work, which covers work associated with nursing, hospital sense, epidemic prevention and control, use of equipment and so on. The daily work of the oral outpatient department carried out by the nursing team guarantees safe, orderly and smooth medical activities. For example, the proper operation of the equipment should be checked: if suction failure occurs during surgery, a patient can be at risk of suffocation; the qualified sterilization of instruments should be ensured: if an instrument is not clean, it can easily cause cross infection between patients; and the standard of the clinical environment should be ensured: if the environment is not up to standard, it can easily cause postoperative infection and other complications [11]. However, due to many daily work items and too many paper records, the work efficiency is low, and some items are even omitted, which virtually increases the workload of the nursing staff.

In March 2021, the supervision group of our hospital inspected the daily work of the outpatient department in the branch and identified many problems, such as an excessive number of daily check-up forms, confusion of the nurses about the daily check-up process, and the omission of daily check-up items. Therefore, to address this problem, our hospital set up a quality improvement team to conduct a status survey and carry out this study. Our aim was to use a daily goals sheet to organize, simplify and make the routine work of the dental clinic more efficient, to evaluate the feasibility, effectiveness and sustainability of using the daily goals sheet in the routine work of the nursing staff and to investigate the satisfaction of the nursing staff with the daily goals sheet.

## Materials and methods

### Study design and situation investigation

The process of study design includes two principles: (1) Plan-Do-Check-Act (PDCA) cycle method. (2) MECE (Mutually exclusive collectively exhaustive) principle. When we created the daily goals sheet for daily work, it was not completed at one time but was combined with the actual situation of the outpatient department; the sheet was constantly improved and optimized by using the PDCA- cycle method. The PDCA cycle, also known as the "quality cycle", is a general management model that originated in the 1920s [12]. The PDCA cycle method is widely used in the total quality management of the scientific path; it can establish the project management objectives and content through the plan, do, check, and action cycle stages to achieve the amplification of system efficiency and improve quality [13, 14]. We followed the MECE principle in the process of making the daily goals sheet. MECE stands for "mutual independence, complete exhaustion, no overlap, and no omission", a thinking principle proposed by McKinsey in the Pyramid Principle. The so-called non-overlap and non-omission means that when a topic is divided into different parts, each part must meet the following requirements [15].

**Fig. 1:**
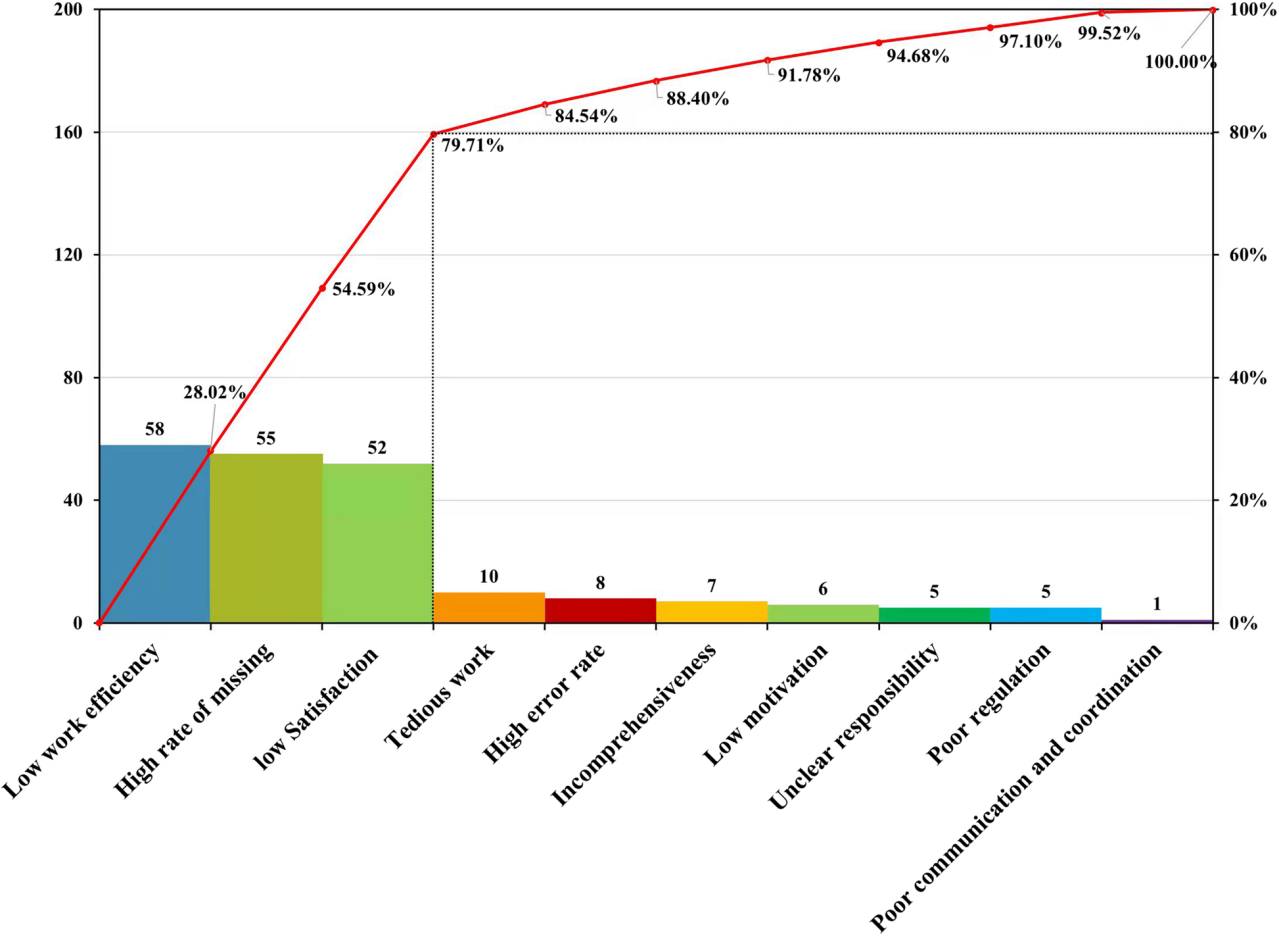
Plato. The main problems in outpatient routine work were identified as low work efficiency, easy omission of inspection items and low satisfaction with original work forms

Situation investigation includes two processes: (1) Brainstorming. (2) Rating problems in the daily work. At present, in view of the problems in the daily work of the outpatient department that were found by the supervision group, all nurses and members of the quality improvement group were organized to conduct a discussion with the theme of "problems existing in the daily work of the outpatient department" by using the brainstorming method [16]. The 10 most popular questions were screened out from many factors and included in a questionnaire, which was voted on by 60 nurses (each of them chose at least one item) to select the main questions. According to the voting results (Table 1) and Pareto's principle, 20% causes 80% of problems and finally makes Plato [17, 18] (Fig. 1). The main problems in outpatient routine work were identified as low work efficiency, the easy omission of inspection items and low satisfaction with the original work forms. Therefore, this paper evaluated the above three problems as the starting point. Our goal was to improve work efficiency, reduce omission rates, and improve nurses' satisfaction with using work forms. The present investigation lays a foundation for the development of this research.

**Table 1:** Proportion of inspection problems

Number	Problem	Frequency	Constituent ratio	Cumulative percentage
1	Low work efficiency	58	28.02%	28.02%
2	High rate of omissions	55	26.57%	54.59%
3	Low Satisfaction	52	25.12%	79.71%
4	Tedious work	10	4.83%	84.54%
5	High error rate	8	3.86%	88.40%
6	Incomprehensiveness	7	3.38%	91.78%
7	Low motivation	6	2.90%	94.68%
8	Unclear responsibility	5	2.42%	97.10%
9	Poor regulation	5	2.42%	99.52%
10	Poor communication and coordination	1	0.48%	100.00%
**Total**		**207**	**100.00%**

### Study settings and ethical issues

This study was carried out in the Shuangxi Outpatient Department of our hospital with a total of 138 staff members, including 70 full-time doctors, 63 full-time nurses and 5 management staff. The study was approved by the Ethics Committee of the School of Stomatology, Shanxi Medical University (No. 2020SLL007). The outpatient routine work daily goals sheet was implemented in our hospital at the beginning of 2021, and the clinical application study of the daily goals sheet and the questionnaire evaluation of nursing staff satisfaction were conducted from May 1, 2021, to May 30, 2021.

### Study subjects

Sixty nurses were randomly selected from all nurses in the outpatient department to participate in this study. The sixty nurses were randomly divided into two groups: the experimental group (*n* = 30) that used the daily goals sheet and the control group (*n* = 30) that used the original form, and the sex, age and working years of the enrolled nurses were statistically analysed (Table 2). Informed consent was obtained from all subjects enrolled in the study. A nursing staff member in the experimental group and a nursing staff member in the control group performed outpatient routine work examinations at the same time every day.

**Table 2:** Basic information of the group members

Group	Sex	Average age	Average years of service(Years)
Test	Female/30	29.00	5.63
Control	Female/30	28.87	5.40

### Safety activities

**Fig. 2:**
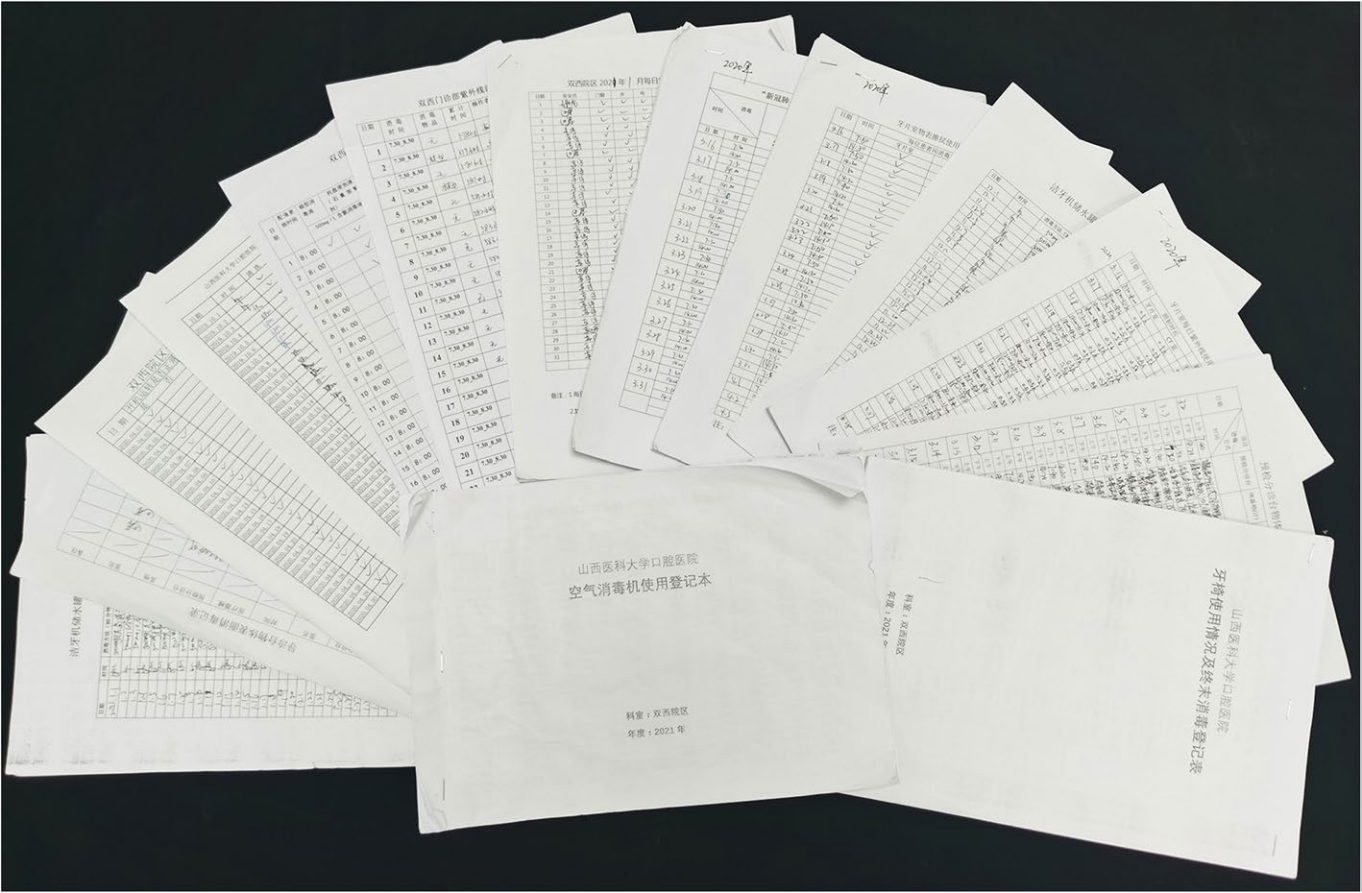
The record sheet applied in our outpatient department before the use of the daily goals sheet. Before the development of the daily goals sheet described in the current study, the completion of recording each daily inspection task required searching through more than 10 other forms, which was a cumbersome process

Before the start of daily diagnosis and treatment activities, the nursing staff should determine the hospital sense and complete equipment inspection and maintenance, epidemic prevention and control to ensure the safety and normal progress of medical activities. The items for routine inspection included the following: air disinfection machine, indoor temperature and humidity, refrigerator temperature and humidity, dental chair and terminal disinfection, soaking liquid (to determine if replacement is needed), ultraviolet disinfection, teeth cleaning machine water storage tank cleaning, leading examining table disinfection, preview triage table disinfection, instrument equipment disinfection, air compressor, video room, ultraviolet disinfection, sewage pump operation, negative pressure pump, pure water machine, and medical staff temperature measurements. Before the development of the daily goals sheet described in the current study, recording the completion of each daily inspection task required searching through more than 10 other forms, which was a cumbersome process. Figure 2 depicts the record sheet applied in our outpatient department before the use of the daily goals sheet. Daily safety activities were carried out by 3–5 nurses, each with several record sheets.

### Development of the daily goals sheet

**Fig. 3:**
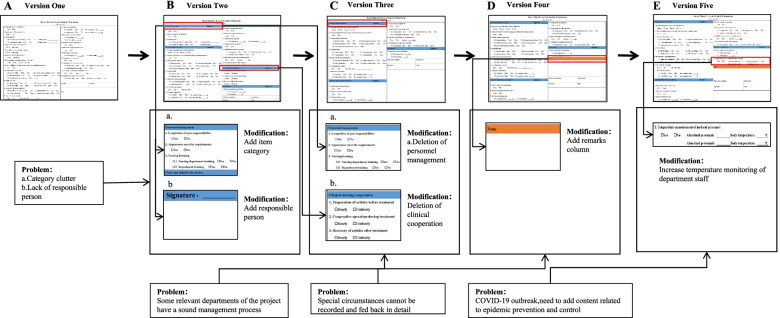
Development process of the daily goals sheet. Under the guidance of the PDCA cycle method, problems were constantly found and resolved. It reflects the sustainability of the daily goals sheet in practice

**Fig. 4:**
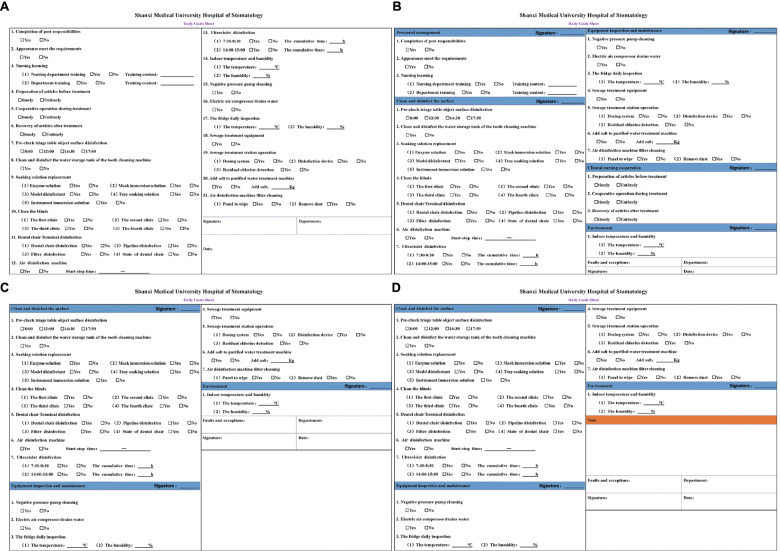
Daily goal sheet evolution process. The first edition of the daily goals sheet contained all the original information (**A**), The second edition added the classification of inspection items and responsible persons (**B**), based on the first edition. In the third edition, some contents were deleted based on the second edition. Since personnel management and clinical cooperation was not within the scope of the routine inspection items, they were deleted (**C**), The fourth edition added a note column based on the third edition to facilitate the recording of problems found (**D**)

**Fig. 5:**
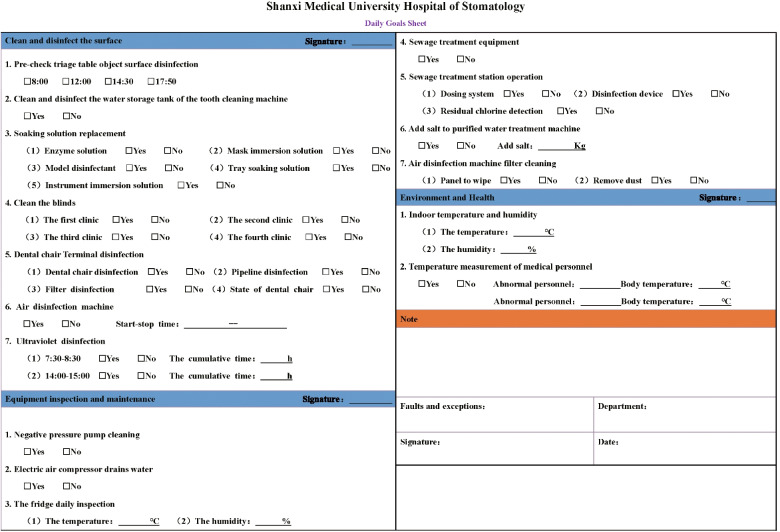
The final version of the daily goals sheet. Due to the impact of the COVID-19 pandemic, the final version of the fourth edition was based on the addition of medical staff temperature monitoring items. The final version is also the version used in the daily clinical work of our hospital, as well as the version used in the efficiency, omission rate and satisfaction survey of this study

We completed 5 versions of the daily goals sheet successively. After the completion of the first version of the daily goals sheet, we randomly selected 10 nurses for the operation and organized the quality improvement team to observe and interview the selected nurses. The 7 professors from the supervision group of our hospital guided the smooth progress of the whole study and ensured the validity and reliability of the whole experiment. Then, the quality improvement team discussed and summarized the experience and updated and improved the daily goals sheet according to the experience. We conducted trial operations for each version, identified the problems, and then carried out the next cycle after improvement. Figure 3 shows the development of the daily goals sheet. Four versions are included in Fig. 4. To give a clear view of our final version, we present it separately (Fig. 5). The final version is also the version used in the daily clinical work of our hospital, as well as the version used in the efficiency, omission rate and satisfaction surveys of this study.

All the items on the daily goals sheet were based on the daily work list, and all users were required to mark "yes" or "no" based on whether each item was completed or was in good working condition. If there were abnormalities or problems related to the inspection items, the problems were traced back and solved in a timely manner, and detailed explanations were provided in the Remarks column of the inspection form. Finally, each user was required to sign and date the inspection form. Notably, the limited paper size limited the number of items on the daily goals sheet; however, to avoid one or more large sheets of paper, which would be inconvenient, the work was sorted, integrated, and ordered to keep the list comprehensive and concise.

### Satisfaction rating method

The questionnaire was divided into five grades: Strongly Agree (5 points), Agree (4 points), Neutral (3 points), Disagree (2 points), and Strongly Disagree (1 point). According to the scoring situation, each question in the questionnaire was statistically analysed.

### Statistical methods

The data results were collated using the spreadsheet program Excel 2007 (Microsoft Corp, Redmond, WA). Statistical analysis was performed using SPSS 22.0 (IBM Germany GmbH, Eningen, Germany) software. The average checking time, omission rate and scores of each question for the experimental group (daily goals sheet group) and control group (original form group) were calculated, and the measurement data are expressed as the mean ± standard deviation. One-way ANOVA was used for comparisons between groups, and a P value less than 0.05 was considered significant. The analyses is descriptive.

## Results

### Experience in the first implementation of the daily goals sheet

After the development of the daily goals sheet, only one nurse needed complete the inspection of all projects; the daily goals sheet was organized and not easy to miss, and it was easier to trace the problems that were found and record the data. The design and production of the daily goals sheet was simple and clear, and the staff participating in the daily check did not complain about it. The overall feedback was that the daily goals sheet was more convenient than the previous record sheet. However, we have summarized our experience below. There was still room for improvement both in the daily goals sheet items and process carried out by the staff.

The daily goals sheet made the our daily inspections easier and more organized, but it also placed higher demands on the staff. The daily goals sheet was designed to maximize convenient labour standards to complete daily inspection work. The original form led to disorderly work, but the new daily goals sheet helped the staff address all items in an orderly and efficient manner. It required every nursing personnel to examine all of the projects and operations and have enough understanding to do so, and such skills can make the whole process smooth, efficient, and high quality.

### Work efficiency and inspection time

We performed statistical analysis on the time spent by the staff in the experimental group and that spent by staff in the control group to complete the outpatient routine work inspection on each of 30 days. The statistical results are shown in Table 3:

**Table 3:** Inspection duration of the experimental group and the control group (min)

**Group**	**D 1**	**D 2**	**D 3**	**D 4**	**D 5**	**D 6**	**D 7**	**D 8**	**D 9**	**D 10**	**D 11**	**D 12**	**D 13**	**D 14**	**D 15**	**D** 16
**Test**	13.5	14.2	14.4	12.8	15.3	13.6	14.3	14.2	15.6	13.1	14.2	13.4	15.0	14.4	15.6	17.6
**Control**	22.6	25.4	24.8	27.5	24.2	28.5	24.5	27.8	25.4	28.6	25.5	26.4	31.0	22.8	20.5	24.3
	**D 17**	**D 18**	**D 19**	**D 20**	**D 21**	**D 22**	**D 23**	**D 24**	**D 25**	**D 26**	**D 27**	**D 28**	**D 29**	**D 30**	**Mean ± SD**	
**Test**	19.2	18.6	15.8	14.9	13.7	18.3	13.5	15.2	16.8	17	16.5	14.5	17.5	14.4	15.2 ± 1.7	
**Control**	21.5	24.3	21	22.8	22.4	27.5	22.5	28.5	23.8	26.3	28.5	26.9	23.4	28.5	25.3 ± 2.7^***^	

*D* represents Day, ***: *P* < 0.001

The average time of the experimental group was 15.20 ± 1.70 min, and the average time of the control group was 25.30 ± 2.70 min, *P* < 0.001.

### Omissions of inspection items

Across the 30 days of data collection, the control group had a total of five nurses with omissions of inspection items, namely, three omissions for the triage surface disinfection inspection and two omissions for the medical personnel's body temperature measurements; the omission rate was 16.67%. The omission rate for the experimental group was 0%.

### Nurse satisfaction

The questionnaire survey results showed that the overall satisfaction of the daily goals sheet of the daily inspection staff was high (Table 4). The staff of the experimental group and the control group scored the 10 questions in the satisfaction questionnaire one by one, and we also collected and statistically analysed the results. The final satisfaction scores for Questions 1–5 and 8–10 in the experimental group were significantly higher than those in the control group, with statistical significance (*P* < 0.001). Only the satisfaction scores of question 6 (my checklist was relatively easier to keep) and question 7 (my workload was reduced) showed no significant difference between the two groups (*P* > 0.05).

**Table 4:** Results of the nursing staff satisfaction questionnaire (points)

**Question**	**Test**		**Control**		**Significance** ***P.* value**
	**Mean**	**SD**	**Mean**	**SD**	
1. I can clearly remember everything I need to check	4.40	0.56	2.60	0.50	< 0.001
2. My inspection items are not easily omitted	4.60	0.50	3.30	0.60	< 0.001
3. I had relatively little time to check	4.53	0.51	1.90	0.55	< 0.001
4. My inspection operation process is relatively convenient	4.47	0.51	2.70	0.53	< 0.001
5. I can complete all the inspection items by myself	3.60	0.67	2.10	0.61	< 0.001
6. My form is relatively easy to keep	3.63	0.49	3.77	0.63	0.362
7. My workload has been reduced	2.53	0.51	2.57	0.50	0.799
8. Work records can be easily searched	4.43	0.57	2.63	0.56	< 0.001
9. I can conduct the inspection in an orderly way, not in a hurry	4.37	0.67	2.07	0.52	< 0.001
10. I can timely and clearly record the problems encountered during the inspection	4.27	0.58	2.97	0.76	< 0.001

## Discussion

The experimental group have less hours at work than the control group, which also showed that the daily goals sheet we created can improve work efficiency.

The results of the omission incidence of the two groups showed that among the 30 nurses in the control group, a total of 5 nurses omitted inspection items, and the omission incidence rate was 16.67%, while that of the experimental group was 0%, indicating that the daily goals sheet could effectively reduce the omission incidence of outpatient routine work inspection items. The occurrence of omissions in medical activities is often serious. For example, an operation can be successfully completed, but negligence in the final counting of instruments can lead to leaving gauze or instruments in a patient's body, which can be dangerous or even fatal [19]. The use of inspection forms has greatly reduced the rate of omissions and the incidence of such medical errors. Similarly, the daily examination work in the oral clinic also plays a role. If a nurse neglects to check the status of equipment, the equipment can fail during an operation, which ultimately leads to medical accidents that we do not want to see. Therefore, the use of the daily goals sheet not only reduces the rate of omission of routine work items but also indirectly improves patient safety. Schmitt scholars came to the same conclusion in their work, and surgical safety checklists in oral and implant surgery can help to make patient treatment safer [5].

The satisfaction results of the two groups for their respective use of the forms showed that the results for Questions 1 and 2 (I can clearly remember every item to be checked, and my inspection items are not easy to omit, *P* < 0.001) were significantly different between the groups. Compared with those in the control group, the staff in the experimental group were more likely to have comprehensive control over the inspection work, and it was not easy to omit any part of the work. The results for Question 3 (my inspection time was relatively small, *P* < 0.001) were significantly different between the groups. Combined with the results in part 3.2, the use of the inspection form led to more time saved and more efficient work. The results of Question 4 (my inspection operation procedure was relatively convenient, *P* < 0.001) were significantly different between the groups. Compared with the inspection daily goals sheet designed for this study, the original process required searching in more than 10 forms for each work inspection item, and all the inspection items were disordered, making the work aimless and inconvenient. The results for Question 5 (I can complete all the examination items by myself, *P* < 0.001) were significantly different between the groups. The new daily goals sheet of inspection items was more concise and logical. According to the overall pattern and design of the outpatient department, all the examination items can be completed by one route from one point in the outpatient department. A single form can cover all the records of the inspection items. With the original record sheet, it might take two or three people to do the job at the same time. The results for Question 6 (my form is easier to save, *P* > 0.05) were not significantly different between the groups. The average score of the experimental group for this item was 3.63 ± 0.49, while that of the control group was 3.77 ± 0.63. Both groups scored higher for this question, although the group using the original form might have scored higher. This result was understandable, as each original form can be used to record a month's work, while one daily goals sheet is produced every day. On an annual basis, the number of daily goals sheets was indeed higher than the number of original forms. However, it simplifies work and improves work efficiency. The results of Question 7 (my workload decreased, *P* > 0.05) were not significantly different between the groups. The average score of the experimental group was 2.53 ± 0.51 and that of the control group was 2.57 ± 0.50. When the daily goals sheet was not used, all the daily inspection work was cooperatively completed by several nurses, and it was necessary to fill in too much examination content on various forms, which wasted time and led to nurses having no clue about the work. After the implementation of the daily goals sheet, all the daily inspection work could be done by a single nurse. Although the inspection items increased, the working hours of each nurse did not increase, the writing content was reduced, and the labour cost was saved. The results for Question 8 (work records can be easily searched, *P* < 0.001) were significantly different between the groups. All the inspection work of each day was recorded in one inspection form, and each form had a record of time. Therefore, no matter whether a certain item or all items in that day were reviewed, the daily goals sheet can be searched quickly. The results for Question 9 (I can conduct the inspection in an orderly way, not in a hurry, *P* < 0.001) were significantly different between the groups. The new daily goals sheet was organized in a logical manner according to the actual working conditions and structure of the outpatient service; the original form was not organized in this manner, resulting in wasted time. The results for Question 10 (I can timely and clearly record the problems encountered during the inspection, *P* < 0.001) were significantly different between the groups. In the design of the inspection form, we left a column for the problems we found and the problems we solved at the end of the form; this structure was more convenient for recording. There is not enough space in the original form to record the problems and rectification measures in detail, which makes it difficult to find and solve the problems.

The daily check table can improve the work efficiency and make the work more convenient at the same time. However, the daily goals sheet has limitations. The first is the preservation of the daily goals sheet. Due to the limited paper size, we could not fully reflect all the inspection items on one piece of paper. As this research advances, we may need to increase the number of pages associated with the inspection form, which brings problems for the daily preservation of the form. In the long run, there will be a large number of daily goal sheets that require more space to keep and are relatively more wasteful of resources. The results of the staff satisfaction questionnaire also identified this problem. Therefore, the next stage of the work plan may be to transition to a paperless process, using tablet computers for inspections, filing, searching, etc., to further improve work efficiency. Additionally, it also better reflects green environmental protection and can save much paper [20]. The second limitation is that although we increased work efficiency, we actually increased the number of checks per nurse in their daily work. Because the initial working mode was completed with the cooperation of several people, each nurse was only responsible for the inspection of a fixed number of items. Now, a staff member is required to complete the work independently in order. Although it can save manpower and improve efficiency, it is associated with higher requirements for professional skills. Each nurse must learn each inspection item, including qualified standards, use and maintenance of equipment and so on. Therefore, we should also ensure opportunities for daily inspection staff members to improve their professional skills, which will not only promote the efficient operation of the daily work of the outpatient department but also enhance the careers of the staff, which is particularly important for individuals.

Compared with medical work, the daily work of oral clinics is equally important, covering nursing, hospital sense, epidemic prevention and control, equipment, etc. The daily work of the oral clinic, carried out by the nursing team, guarantees safe and orderly medical activities. The operation of medical equipment, storage of medical drugs, sterilization of medical equipment and clean diagnosis and treatment environments will affect the safety of patients and wound healing. We believe that the daily goals sheet not only improves the efficiency and quality of daily work but also has a positive impact on the prognosis of patients. Therefore, the research group will consider the influence of the outpatient daily goals sheet on patient prognosis as the content of the next study.

Reviewing the entire study process, it was found that the daily goals sheet improved the work efficiency of outpatient nurses and saved labour and time costs. At the same time, we also, under the guidance of the PDCA cycle, constantly found problems, corrected problems, and achieved sustainable development. For individual nurses, the new working mode improved their job satisfaction and enthusiasm. The daily goals sheet cannot remain unchanged, and it would need to be adjusted any time we find a problem in our daily work or updated projects. Therefore, this paper only provides a new idea for our daily outpatient work; we still need to combine our actual situation, and we can design a suitable daily goals sheet under the guidance of the PDCA cycle method.

## Conclusion

The daily goals sheet designed and produced by us makes the daily work of the dental clinic more efficient and convenient. In this paper, the daily goals sheet improves work efficiency, reduces work omission rate and improves employee satisfaction. Since there are few reports regarding the routine work of oral outpatient departments or the daily goals sheet of medical work, we hope this article provides a new working idea for other medical professionals. However, the daily goals sheet was designed according to our actual situation and needs, and others are encouraged to use the PDCA cycle method to produce a unique daily goals sheet through continuous improvement.

## Data Availability

The datasets used and/or analysed during the current study are available from the corresponding author on reasonable request.

## References

[1] Haynes AB, Weiser TG, Berry WR, et al. Safe surgery saves lives study group. A surgical safety checklist to reduce morbidity and mortality in a global population. N Engl J Med. 2009; 360: 491–9. 10.1056/NEJMsa0810119 19144931

[2] WHO Guidelines for Safe Surgery (2009). Safe Surgery Saves Lives.

[3] Weiser TG, Haynes AB, Dziekan G (2010). Effect of a 19-item surgical safety checklist during urgent operations in a global patient population. Ann Surg.

[4] Thusu S, Panesar S, Bedi R (2012). Patient safety in dentistry - state of play as revealed by a national database of errors. Br Dent J.

[5] Schmitt CM, Buchbender M, Musazada S, Bergauer B, Neukam FW (2018). Evaluation of Staff Satisfaction After Implementation of a Surgical Safety Checklist in the Ambulatory of an Oral and Maxillofacial Surgery Department and its Impact on Patient Safety. J Oral Maxillofacial Surg.

[6] Pronovost PJ, Goeschel CA, Colantuoni E (2010). Sustaining reductions in catheter related bloodstream infections in Michigan intensive care units: observational study. BMJ.

[7] Holzmueller CG, Timmel J, Kent PS (2009). Implementing a team-based daily goals sheet in a non-ICU setting. Jt Comm J Qual Patient Saf.

[8] Fitzpatrick G, Ellingsen G (2013). A review of 25 years of CSCW research in healthcare: Contributions, challenges and future agendas. CSCW J.

[9] Aspesi AV, Kauffmann GE, Davis AM (2013). IBCD: development and testing of a checklist to improve quality of care for hospitalized general medical patients. Jt Comm J Qual Patient Saf.

[10] Arora VM, McGory ML, Fung CH (2007). Quality indicators for hospitalization and surgery in vulnerable elders. J Am Geriatr Soc.

[11] Johansson G. Health-related research to improve working environment and patient safety (presentation)[J]. Nordic Ergon Hum Factors Soc. 2013;13:34–42.

[12] Shewhart WA. Economic Control of Quality of Manufactured Product. Am Math Mon. 1933;40(6):353–55.

[13] Petersen PB (1993). The New Economics for Industry, Government. Education Acad Manag Exec.

[14] Gu S, Zhang A, Huo G (2021). Application of PDCA cycle management for postgraduate medical students during the COVID-19 pandemic. BMC Med Educ.

[15] Lee CY, Chen BS. Mutually-Exclusive-and-Collectively-Exhaustive Feature Selection Scheme[J]. Appl Soft Comput. 2017;4:11–22.

[16] Dugosh KL, Paulus PB, Roland EJ (2000). Cognitive stimulation in brainstorming. J Pers Soc Psychol.

[17] Engle P. The 80–20 rule's guide to success. Ind Eng. 2015;47(2):20.

[18] Landauer, Dumais TK, Susan T. A solution to Plato's problem: The latent semantic analysis theory of acquisition, induction, and representation of knowledge. Psychol Rev. 1997;104(2):211.

[19] Jiang-ping G, Xiao-xiong W (1998). Imaging diagnosis of iatrogenic residual gauze in vivo. J Med Imag.

[20] Amin M B , Adhimy S . Pedatren: manajemen pesantren berbasis paperless office. re-JIEM. Res J Islamic Educ Manage. 2020; 3: 52.

